# Roflumilast and aquaporin‐2 regulation in rat renal inner medullary collecting duct

**DOI:** 10.14814/phy2.13121

**Published:** 2017-01-20

**Authors:** Ezigbobiara N. Umejiego, Yanhua Wang, Mark A. Knepper, Chung‐Lin Chou

**Affiliations:** ^1^ Epithelial Systems Biology LaboratorySystems Biology CenterNHLBINational Institutes of HealthBethesdaMaryland20892‐1603; ^2^ Renal DivisionDepartment of MedicineEmory UniversityAtlantaGeorgia30322

**Keywords:** cAMP phosphodiesterase, kidney, nephrogenic diabetes insipidus, X chromosome

## Abstract

Roflumilast is a cyclic nucleotide phosphodiesterase inhibitor that is FDA‐approved for treatment of chronic obstructive pulmonary disease. With a view toward possible use for treatment of patients with X‐linked nephrogenic diabetes insipidus (NDI) due to hemizygous mutations in the V2 vasopressin receptor, this study sought to determine the effect of roflumilast on aquaporin‐2 (AQP2) phosphorylation, AQP2 trafficking, and water permeability in the rat inner medullary collecting duct (IMCD). In the presence of the vasopressin analog dDAVP (0.1 nmol/L), both roflumilast and its active metabolite roflumilast *N*‐oxide (RNO) significantly increased phosphorylation at S256, S264, and S269, and decreased phosphorylation at S261 (immunoblotting) in IMCD suspensions in a dose‐dependent manner (3–3000 nmol/L). Another commonly used phosphodiesterase inhibitor, IBMX, affected phosphorylation only at the highest concentration in this range. However, neither roflumilast nor RNO had an effect on AQP2 phosphorylation in the absence of vasopressin. Furthermore, roflumilast alone did not increase AQP2 trafficking to the plasma membrane (immunofluorescence) or increase water permeability in freshly microdissected perfused IMCD segments. We conclude that roflumilast can be used to enhance vasopressin's action on AQP2 activity in the renal collecting duct, but has no detectable effect in the absence of vasopressin. These findings suggest that roflumilast may not have a beneficial effect in X‐linked NDI, but could find useful application in acquired NDI.

## Introduction

X‐linked nephrogenic diabetes insipidus (NDI) is a rare genetic disorder that is associated with marked polyuria due to mutations in the gene that codes for the V2 vasopressin receptor (gene symbol: *Avpr2*) (Bockenhauer and Bichet 2015)(Boone and Deen 2008). This receptor normally couples to the heterotrimeric G‐protein G*α*s and activates adenylyl cyclases in the renal collecting duct. Upon stimulation by binding of vasopressin, the receptor‐mediated signaling elicits increases in intracellular cyclic AMP (cAMP) and secondary increases in intracellular calcium (Star et al. 1988; Yip 2002). These responses regulate the molecular water channel aquaporin‐2 (AQP2), which is rate‐limiting for transepithelial water transport in the collecting duct epithelium. AQP2 is regulated in the short term by phosphorylation changes. Vasopressin regulates phosphorylation of AQP2 at four serine residues (S256, S261, S264, and S269) located at the carboxyl terminus (Hoffert et al. 2006; Yu et al. 2009). While phosphorylation changes at S256 are necessary for exocytosis of AQP2 (Fushimi et al. 1997; Kamsteeg et al. 2000), phosphorylation at S261 and S269 controls AQP2 endocytosis (Hoffert et al. 2007, 2008; Moeller et al. 2008). These regulatory processes allow AQP2 to play a central role in the urine concentration mechanism of the kidney.

With a defective V2 receptor in X‐linked NDI (Bichet et al. 1993; Holtzman et al. 1993a,b; Moses and Scheinman 1993), other means of regulating AQP2 that bypasses this receptor offer the hope of alleviating and/or treating the symptoms that accompanies this malady. It may be possible to ameliorate the disease through the use of agents that can either activate other G*α*s‐linked receptors in collecting duct cells (Bouley et al. 2000, 2011; Li et al. 2009; Olesen et al. 2011; Procino et al. 2014) or that can increase cAMP signaling by other means (Bouley et al. 2005; Li et al. 2011; Procino et al. 2014; Russo et al. 2006). Although economic driving forces are not conducive to de novo development of drugs to treat X‐linked NDI, it is possible that drugs developed and FDA‐approved for treatment of other diseases could be employed cross‐label for its treatment. One such drug with potential for treatment of X‐linked NDI is roflumilast, a FDA‐approved agent for treatment of severe chronic obstructive pulmonary disease (COPD) (Beghe et al. 2013; Rabe 2010, 2011). Roflumilast is a cyclic nucleotide phosphodiesterase (PDE) inhibitor that is selective for type 4 PDEs. PDE4 is coded by four different genes, viz. *Pde4a, Pde4b, Pde4c,* and *Pde4d*, and is one of three cAMP‐specific PDEs (with PDE7 and PDE8) expressed in the kidney inner medullary collecting duct (Beavo 1995; Bolger 1994; Conti et al. 1995, 2003; Dousa 1999; Engels et al. 1994; Francis et al. 2011; Maurice et al. 2014; McSorley et al. 2006; Stefan et al. 2007; Uawithya et al. 2008). Accordingly, the PDE inhibitor roflumilast could theoretically ameliorate X‐linked NDI by increasing intracellular cAMP in collecting duct cells, despite the absence of a functional V2 vasopressin receptor.

In this study, we do the groundwork to address the feasibility of such an approach, working with isolated inner medullary collecting ducts (IMCDs) from rats. We carry out experiments to test the effects of roflumilast on: (1) phosphorylation of AQP2 at the four vasopressin‐regulated sites (S256, S261, S264, and S269), (2) trafficking of AQP2 to the apical plasma membrane in collecting duct cells, and (3) osmotic water permeability in isolated perfused IMCD segments.

## Methods

### Rats

Untreated Sprague–Dawley rats were used for harvest of renal tissue as described previously (Miranda et al. 2014). Animal procedures were approved by the National Heart, Lung, and Blood Institute Animal Care and Use Committee (protocol no. H‐0110R3). Briefly, pathogen‐free male Sprague–Dawley rats (Taconic Farms, Germantown, NY), weighing 190–250 g, were used for these studies. Rats were maintained on autoclaved standard rat diet and ad libitum drinking water in filter‐top microisolator cages. The animals received an intraperitoneal injection of furosemide (0.50 mg; Hospira, Lake Forest, IL) on the experimental day, to trigger a rapid diuresis and clear out most of the inner medullary axial solute concentration gradient present in untreated animals (Sands and Knepper 1987). After 20–25 min, the animals were euthanized by decapitation without anesthesia. Kidneys were rapidly removed and chilled on ice‐cold normal saline. The inner medulla tissues were subsequently processed for one of three purposes summarized below: (1) preparing inner medullary collecting duct (IMCD) suspensions for immunoblotting; (2) measuring water transport in isolated, perfused tubules; or (3) microdissecting of single IMCD tubules for immunofluorescence.

### Preparation of rat kidney IMCD suspensions

IMCD suspensions were prepared as previously described (Miranda et al. 2014). Briefly, kidney inner medullas were dissected, minced, and digested by incubation at 37°C for 70–90 min in digestion solution (250 mmol/L sucrose, 10 mmol/L triethanolamine, pH 7.6) containing collagenase B (3 mg/mL; Roche, Indianapolis, IN) and hyaluronidase (1400 U/mL; Worthington, Lakewood, NJ). The suspensions were centrifuged (70 g, 20 sec), and the supernatant was discarded. The pellet was washed and resuspended with IMCD (bicarbonate‐buffered) solution that had been preequilibrated with 95% air/5% CO_2_ (repeated twice). The composition of the IMCD solution was (in mmol/L) 118 NaCl, 25 NaHCO_3_, 5 KCl, 4 Na_2_HPO_4_, 2 CaCl_2_, 1.2 MgSO_4_, 5.5 glucose, and 5 Na acetate (290 mosmol/kg H_2_O). IMCD suspensions were allowed to equilibrate with gentle stirring with the aid of a microstirring bar at 37°C with 95% air/5% CO_2_ supply for 10 min before use. Samples were incubated with roflumilast (product no. S2131, Selleck Chemicals), roflumilast *N*‐oxide (RNO; product no. R639710, Toronto Research Chemicals), or arginine vasopressin (product no. H1780, Bachem) as described in the figure legends. Upon completion of the incubation, the suspensions were pelleted by centrifugation at 16,000 *g* for 2 min to harvest the IMCD segments. Pellets were resuspended in 1X Laemmli buffer (1.5% SDS, 10 mmol/L Tris, pH 6.8) containing Halt protease and phosphatase inhibitors (Thermo Scientific, Rockford, IL) and passed through a DNA shredder (Qiagen, Valencia, CA) for 1 min. Protein concentration was determined by the BCA method (Thermo Scientific). Samples were diluted to a protein concentration of 2 mg/mL, glycerol 6%, and DTT 40 mmol/L. Samples were heated at 60°C for 10 min prior to storing at 4°C.

Before immunoblotting, samples were separated on an SDS‐PAGE gel (12% Tris‐Glycine, precast polyacrylamide, Criterion TGX, Bio‐Rad) and stained with Coomassie blue to check for equal loading (Kim et al. 1999). This “total‐protein‐profile” approach has been the standard for equal‐loading controls for vasopressin studies in our laboratory, because of studies showing that vasopressin alters the abundances of single proteins frequently used as loading controls, viz. GAPDH (Pisitkun et al. 2006), beta‐actin (Pisitkun et al. 2006), and gamma‐actin (van Balkom et al. 2004).

Immunoblotting was performed as previously described (Miranda et al. 2014). Proteins were resolved by SDS‐PAGE on 12% Tris‐Glycine polyacrylamide gels (Criterion TGX, Bio‐Rad) and transferred electrophoretically onto 0.2 *μ*m nitrocellulose membranes (Bio‐Rad, #162‐0112). Membranes were blocked for 1 h with Odyssey blocking buffer (Li‐Cor, Lincoln, NE), rinsed, and probed overnight at 4°C with the respective affinity‐purified antibodies at an appropriate dilution in Odyssey blocking buffer containing 0.1% Tween 20. After 1 h incubation with secondary antibody (Alexa Fluor 680‐nm goat anti‐rabbit immunoglobulin G; Invitrogen, Carlsbad, CA) at 1:5,000 dilution, sites of antibody–antigen reaction were detected and quantified using an Odyssey infrared imager (Li‐Cor). Band densities were normalized and analyzed statistically as described in the figure legends.

### Tubule perfusion

IMCD segments were dissected from the kidneys of pathogen‐free male Sprague–Dawley rats weighing 100–200 g using methods previously described (Chou et al. 2008). Briefly, coronal slices containing the inner medulla were cut from the kidneys and were transferred to a dissection dish containing chilled dissection solution (12°C) for isolation of collecting ducts. Terminal IMCDs were dissected using a Wild M8 dissection microscope. The dissecting solution contained the following (in mmol/L): 120 NaCl, 25 NaHCO_3_, 2 CaCl_2_, 2 K_2_HPO_4_, and 1.2 MgSO_4_, 5.5 glucose. This solution was suffused continuously with 95% air/5% CO_2_ before and during the dissection.

The microdissected terminal IMCDs were mounted on concentric pipettes and perfused in vitro at 37°C as previously described (Chou et al. 2008). The perfusion and bath solutions were identical to the dissection medium, except that an additional 111 mmol/L NaCl was added to the bath solution to create a 200 mOsm transepithelial osmotic gradient (490 vs. 290 mOsm). The osmolalities of the perfusate and bath were measured by vapor pressure osmometry (Wescor, Logan, UT), allowing the calculation of the actual osmolality gradient. One millimolar fluorescein sulfonate (Molecular Probes) was added to the luminal perfusate as a readily measurable, impermeant luminal marker, which is concentrated when water moves from the lumen to the peritubular bath. Fluorescein sulfonate concentrations in perfusate and collected fluid were measured by continuous‐flow ultramicrofluorometry (Wall et al. 1992), allowing calculation of transepithelial water flux and osmotic water permeability (P_f_) according to the equation of Al‐Zahid et al. (1977).

### Immunofluorescence labeling of microdissected renal IMCDs

Tubules were microdissected as described above except that a HEPES‐buffered solution was used, composed of (in mmol/L): 118 NaCl, 10 Hepes, 5 KCl, 2.5 Na_2_HPO_4_, 2 CaCl_2_, 1.2 MgSO_4_, 5.5 glucose, and 5 Na acetate (pH 7.4). The microdissected IMCD segments were immobilized on glass bottomed culture dishes (MatTek, #P35GC‐1.5‐14, Ashland, MA) coated with Cell Tak (Corning, #354240, Bedford, MA). The tubules were incubated with or without an agent (dDAVP and/or roflumilast) for 30 min at 37°C and then immediately fixed with 4% paraformaldehyde for 20 min at room temperature, rinsed twice with Dulbecco's PBS with Ca^2+^ and Mg^2+^ (GIBCO, #14040‐133), followed with fixative removal using 100 mmol/L glycine for 20 min. The tubules were washed again with PBS three times followed by incubation in 100% methanol at −20°C for 20 min. On completion of the methanol treatment, tubules were washed for 5 min with PBS. Tubules were permeabilized using 0.3% Triton X‐100/0.1% BSA/PBS for 10 min, followed with three PBS washes, and blocking (30 min) with 1% BSA/0.2% gelatin. IMCDs were incubated overnight (4°C) in AQP2 antibody (Ab K5007) at an IgG concentration of 17.5 ng/mL or an antibody to AQP1 (L266) at 17.2 ng/mL. Samples were washed three times with 0.05% Tween‐20 in PBS for 10 min each, followed with a 2 h incubation with the secondary antibody (1:2,000 dilution, Alexa Fluor 488 goat anti‐rabbit immunoglobulin G; Invitrogen, Carlsbad, CA) at room temperature. To label nuclei, tubules were incubated with DAPI (Molecular Probes, D3571, 1:250 dilution in PBS) for 10 min, followed by washing with 0.05% Tween‐20/PBS for 10 min (three times). This was followed with a quick wash with PBS (three times), and imaging using confocal microscopy (Zeiss LSM 780).

### Antibodies

All primary antibodies used in this study are previously characterized affinity‐purified rabbit polyclonal antibodies. The holoprotein‐directed antibody, AQP2 (Hoffert et al. 2008), was used for both immunoblotting and immunofluorescence. The following phospho‐specific antibodies were used for immunoblotting: phospho‐S256‐AQP2 (Xie et al. 2010), phospho‐S261‐AQP2 (Hoffert et al. 2007), phospho‐S264‐AQP2 (Hoffert et al. 2008), and phospho‐S269‐AQP2 (Hoffert et al. 2008). A rabbit polyclonal antibody to AQP1 (Terris et al. 1996) was used for immunofluorescence controls.

### Statistics

All data were presented as means ± SE. The *P* value of Student *t*‐test < 0.05 was considered statistically significant.

## Results

### Effect of roflumilast on aquaporin‐2 phosphorylation in renal IMCDs

**Figure 1:**
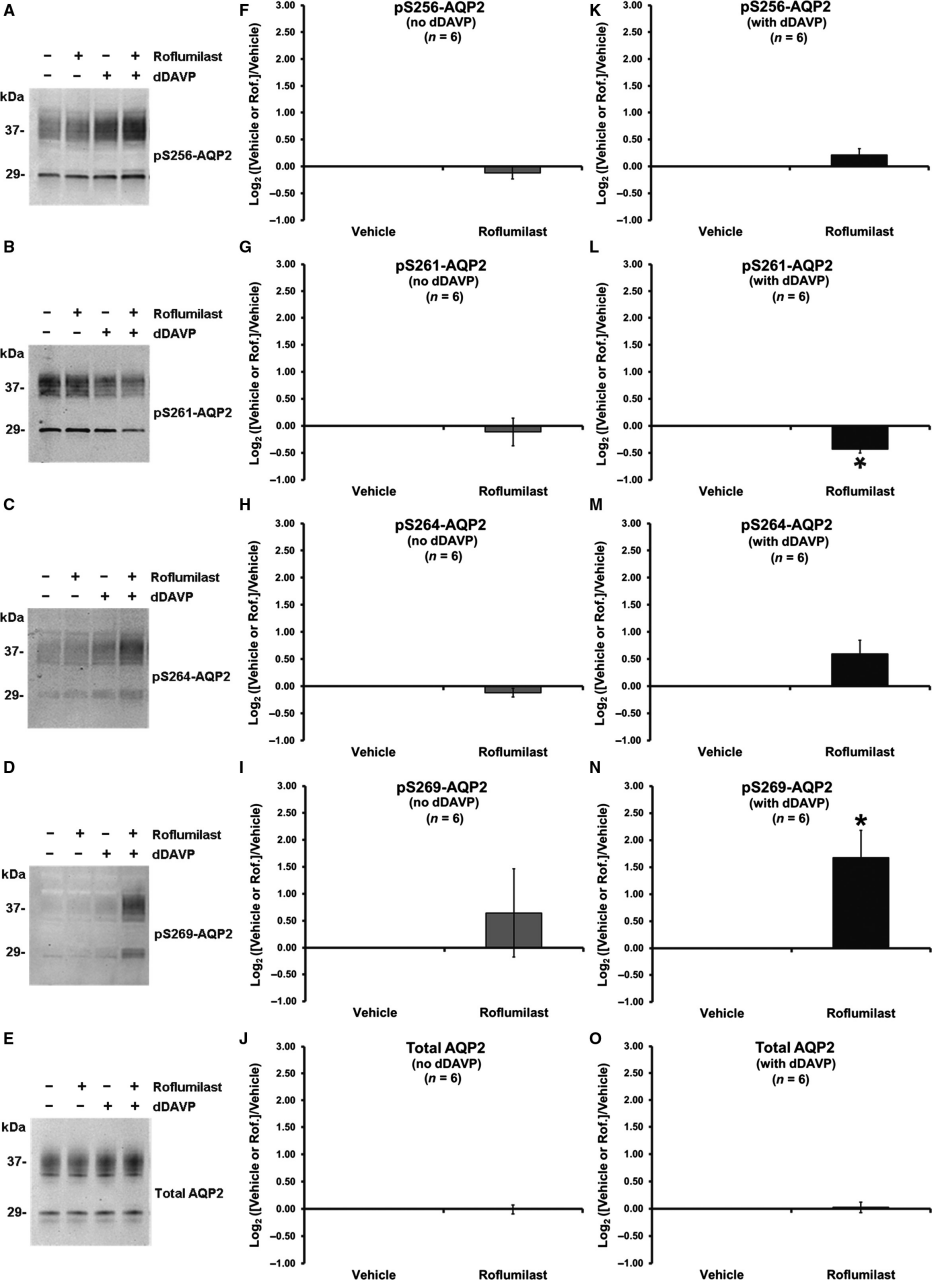
Effect of roflumilast on AQP2 phosphorylation changes in rat IMCD suspensions. IMCD suspensions were incubated with vehicle or roflumilast (30 nmol/L) in the absence or presence of 0.1 nmol/L dDAVP for 30 min. (A–E) immunoblots for the four vasopressin‐regulated serine sites (S256, S261, S264, and S269) of AQP2 and total AQP2. (F–O) band density analysis depicted as log_2_ value of the effect of vehicle or roflumilast normalized by vehicle in the absence (F–J) or presence (K–O) of dDAVP. *Statistically significant (*P* < 0.05). IMCD, inner medullary collecting duct.

Vasopressin increases phosphorylation of AQP2 at three serines in its COOH‐terminal tail (S256, S264, and S269) and decreases phosphorylation at S261 (Hoffert et al. 2006; Yu et al. 2009). Phosphorylation changes at these sites have been shown to serve an integral role in the recycling and trafficking of AQP2, to and from the apical plasma membrane, and thus its short‐term regulation of water permeability (Hoffert et al. 2008; Moeller et al. 2009). As such, our study began by measuring AQP2 phosphorylation changes at S256, S261, S264, and S269 in response to roflumilast (at the therapeutic concentration, 30 nmol/L (Lambert et al. 2014)) in IMCD suspensions from rats (Fig. 1). The left‐hand column (Fig. 1A–E) shows examples of the immunoblots. AQP2 is seen as two bands, a lower 29 kDa nonglycosylated band and an upper 33–40 kDa glycosylated band. (AQP2 tetramers contain both forms (Hendriks et al. 2004)). To quantify the immunoblots, we measured total density of both bands. The middle column (Fig. 1F–J) shows summary graphs of the effects of roflumilast on band densities in the absence of dDAVP. As can be seen, roflumilast had no effect in the absence of the vasopressin analog. The last column (Fig. 1K–O) shows summary graphs of the effects of roflumilast in the presence of dDAVP (at a concentration that produces a maximal effect on water permeability (Star et al. 1988), 0.1 nmol/L). As can be seen, in the presence of dDAVP, roflumilast significantly decreased phosphorylation of S261, while markedly increasing phosphorylation at S269.

### Effect of roflumilast *N*‐oxide on aquaporin‐2 phosphorylation in IMCD cell suspensions

**Figure 2:**
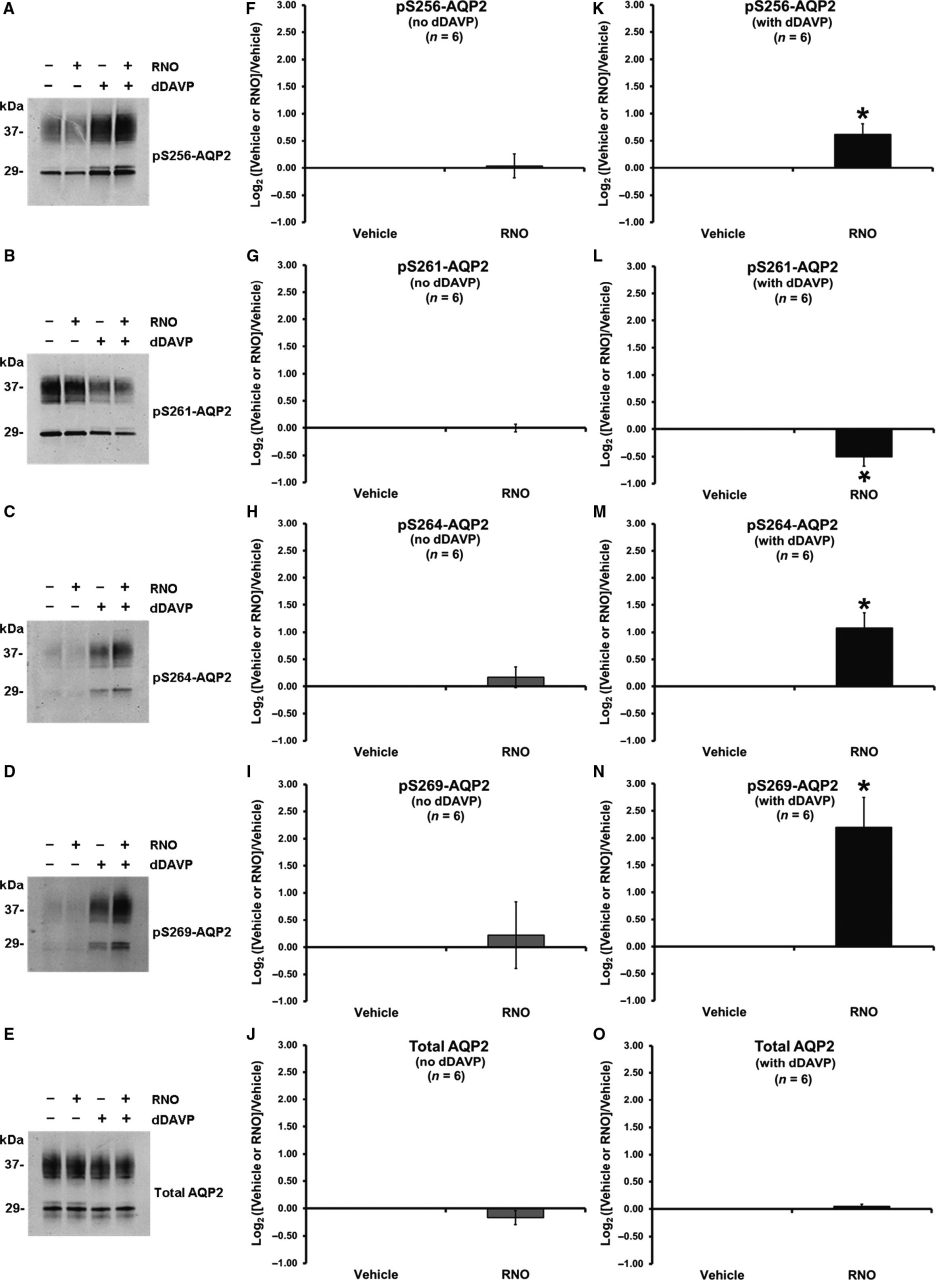
Effect of roflumilast *N*‐oxide on AQP2 phosphorylation changes in rat IMCD suspensions. IMCD suspensions were incubated with vehicle or RNO (30 nmol/L) in the absence or presence of 0.1 nmol/L dDAVP for 30 min. (A–E) immunoblots for the four vasopressin‐regulated serine sites (S256, S261, S264, and S269) of AQP2 and total AQP2. (F–O) band density analysis depicted as log_2_ value of the effect of vehicle or RNO normalized by vehicle in the absence (F–J) or presence (K–O) of dDAVP. *Statistically significant (*P* < 0.05). IMCD, inner medullary collecting duct; RNO, roflumilast *N*‐oxide.

It could be argued that roflumilast had no effect in the absence of dDAVP because the conversion of roflumilast to its pharmacologically active form, RNO (Hatzelmann and Schudt 2001), may not be optimal in collecting duct cells. Therefore, we carried out studies like those described in the previous paragraph to test the effects of RNO on AQP2 phosphorylation changes at S256, S261, S264, and S269 (Fig. 2). As was seen with roflumilast, 30 nmol/L RNO produced no effects on AQP2 phosphorylation in the absence of dDAVP. However, in the presence of dDAVP, RNO produced a strong effect on AQP2 phosphorylation at all four vasopressin‐regulated sites, significantly increasing phosphorylation at S256, S264, and S269, while significantly decreasing phosphorylation at S261. Thus, RNO accentuated the phosphorylation effects of dDAVP at all sites. Taken together, however, the data in Figures 1 and 2 do not support the idea that roflumilast has effects that are independent of vasopressin V2 receptor occupation in the renal collecting duct, although the agent enhanced responses to vasopressin.

### Dose–response comparison of roflumilast and IBMX effects on aquaporin‐2 phosphorylation in the presence of vasopressin

**Figure 3:**
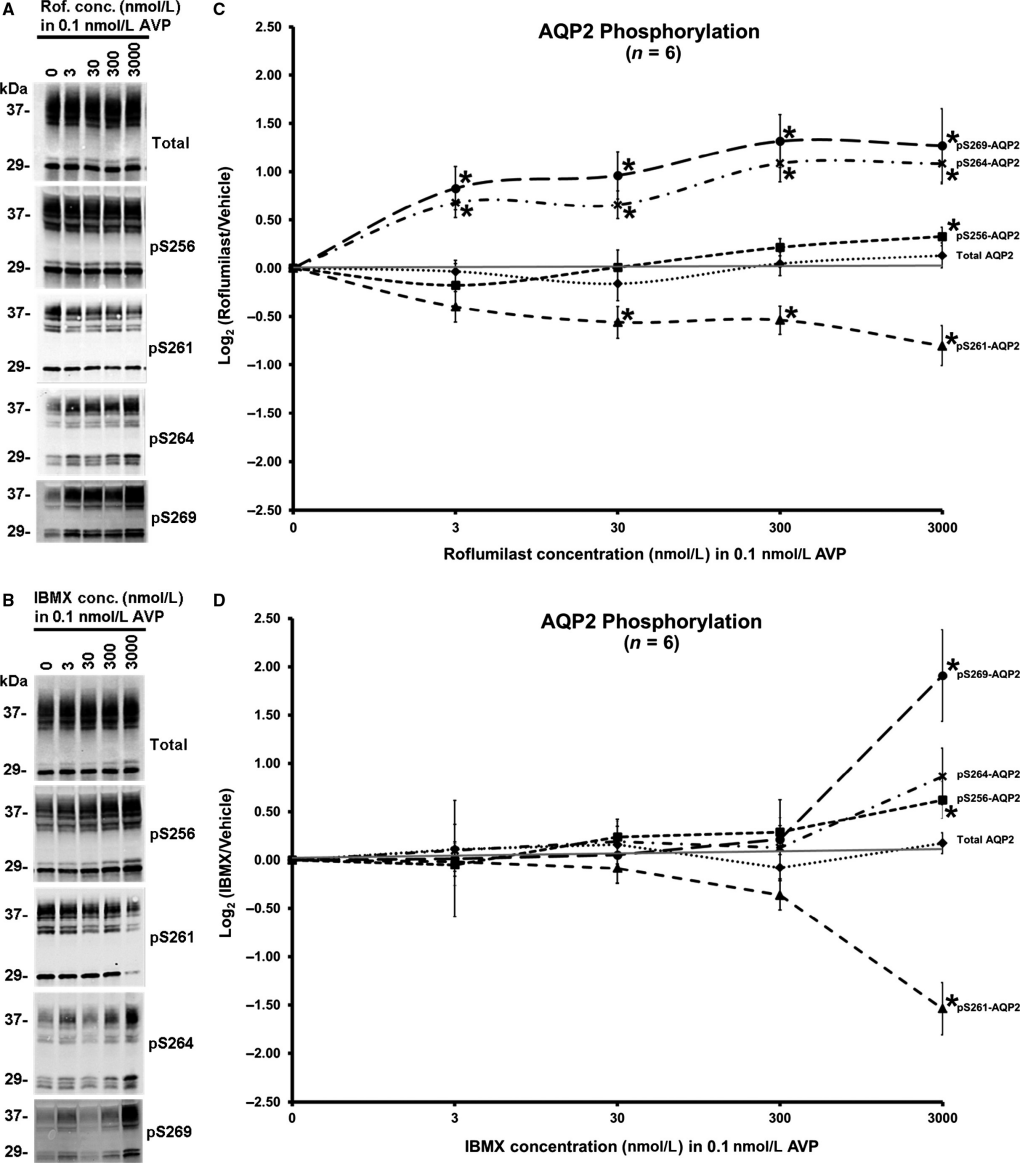
Dose‐dependent effect of roflumilast and IBMX on AQP2 phosphorylation changes in IMCD suspensions in the presence of 0.1 nmol/L AVP. The dose range (3 nmol/L, 30 nmol/L, 300 nmol/L, and 3000 nmol/L) and the incubation time (30 min) were the same for both agents. (A&B) immunoblots for total AQP2 and the four vasopressin‐regulated serine sites (S256, S261, S264, and S269) of AQP2. (C&D) line graphs summarizing the changes in normalized band densities of the total and four phosphorylation sites of AQP2 elicited by roflumilast (C) or IBMX (D). *n* = 6 for both groups. *Statistically significant (*P* < 0.05). IBMX, isobutyl‐1‐methylxanthine; IMCD, inner medullary collecting duct.

Since 3‐isobutyl‐1‐methylxanthine (IBMX) is a nonselective phosphodiesterase inhibitor commonly used in renal physiology experiments (Bolger 1994), we next compared the efficacy of roflumilast and IBMX in inducing AVP‐stimulated AQP2 phosphorylation. Figure 3 shows dose–response data on the effects of roflumilast and IBMX. AQP2 phosphorylation increases were evident from the lowest to highest dose of roflumilast for S256, S264 and S269, while there were progressive decreases in phosphorylation at S261. However, these phosphorylation changes were minimal in response to IBMX at all but the highest concentration, 3000 nmol/L (Fig. 3D). Thus, roflumilast is much more potent than IBMX in increasing vasopressin‐induced AQP2 phosphorylation.

### Effect of roflumilast on aquaporin‐2 phosphorylation in the presence of EP4 PGE_2_ Receptor Agonist, ONO‐AE1‐329 (ONO)

**Figure 4:**
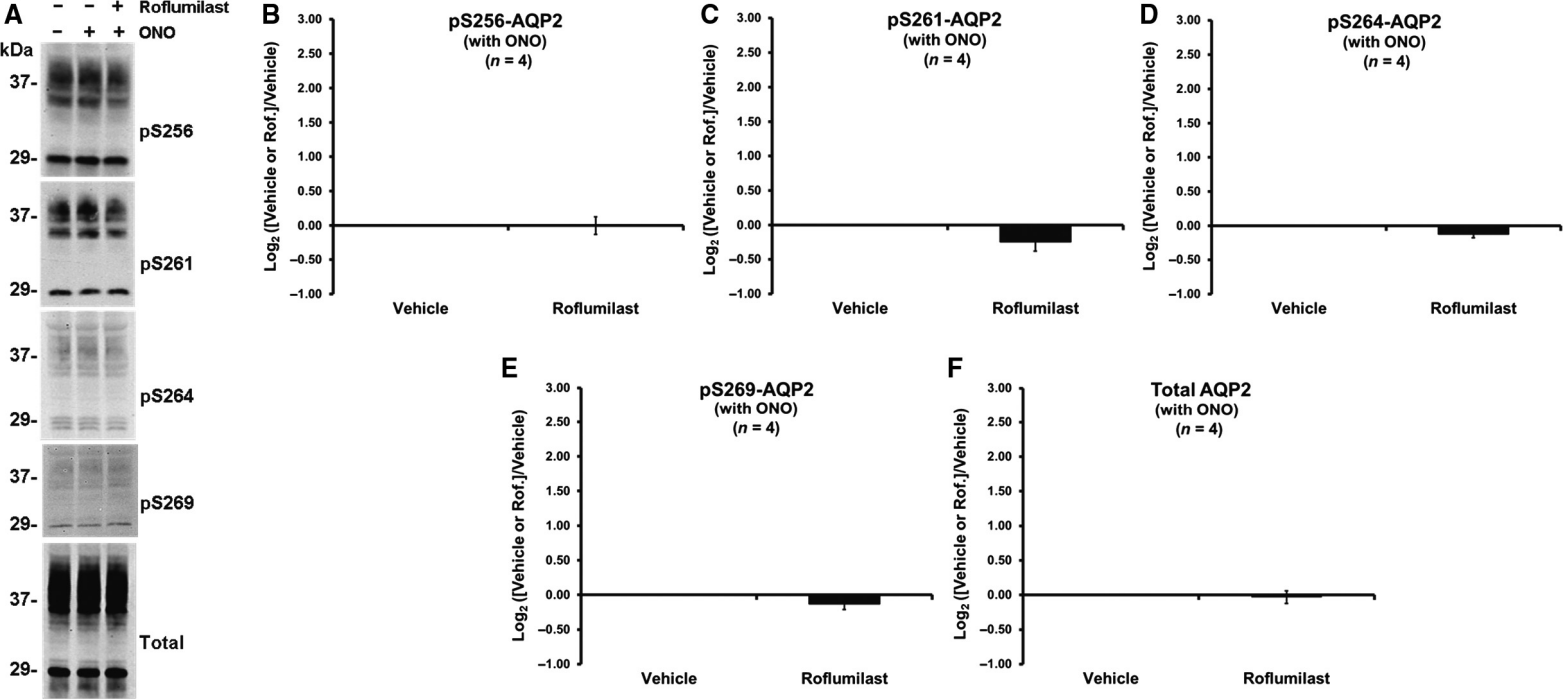
Effect of roflumilast on AQP2 phosphorylation changes in rat IMCD suspensions in the presence of a selective EP4 agonist ONO‐AE1‐329 (30 min incubation period). (A) immunoblots for the four vasopressin‐regulated serine sites (S256, S261, S264, and S269) of AQP2 and total AQP2. (B–F) bar graphs of the normalized band density analysis. IMCD, inner medullary collecting duct.

In a previous study, we showed in mouse cortical collecting ducts that ONO at concentration of 100 nmol/L increased the osmotic water permeability, presumably by activating the EP4 PGE2 receptor (Li et al. 2009). This increase in osmotic water permeability is thought to be mediated through cAMP actions. We hypothesize that the combination of ONO and roflumilast could have a significant effect on AQP2 phosphorylation. We tested this in IMCD suspensions (Fig. 4). However, there was no significant effect of ONO alone (100 nmol/L) or the combination of ONO and roflumilast (30 nmol/L) on AQP2 phosphorylation at any of the four vasopressin‐regulated sites.

### Effect of roflumilast on aquaporin‐2 trafficking in microdissected inner medullary collecting ducts

**Figure 5:**
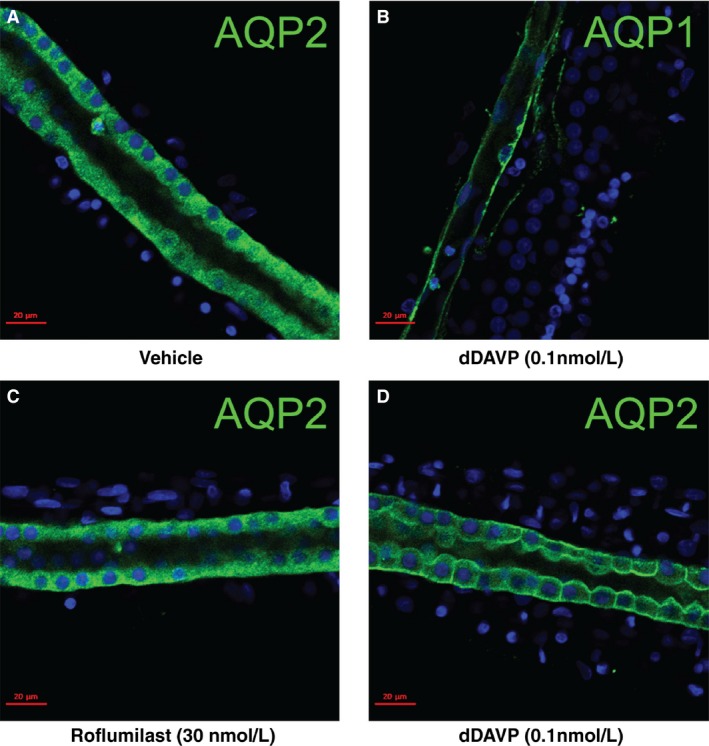
Immunofluorescence labeling of AQP2 in microdissected IMCD segments. Freshly microdissected IMCDs were incubated with vehicle (A) 30 nmol/L roflumilast (C) or 0.1 nmol/L dDAVP (D) for 30 min, followed by fixation and immunofluorescence staining for AQP2. B, using an AQP1 antibody at a concentration similar to that used for AQP2 antibody in A, C and D, AQP1 identified only the thin structures attached to an IMCD segment (pretreated with 0.1 nmol/L dDAVP) but not IMCD. Green: AQP2 or AQP1, blue: DAPI. (*n* = 3). IMCD, inner medullary collecting duct.

Vasopressin regulates the water permeability of collecting duct cells in part by regulating AQP2 trafficking to and from the apical plasma membrane in collecting duct cells (Brown 2003; Nielsen et al. 1995). In theory, this trafficking could be in part independent of AQP2 phosphorylation. To test the ability of roflumilast to redistribute AQP2 to the apical plasma membrane, we exposed microdissected IMCD segments in vitro to roflumilast (30 nmol/L) or dDAVP (0.1 nmol/L). After incubation, the tubules were fixed and labeled for immunocytochemistry. Figure 5 shows representative confocal images. dDAVP caused strong redistribution of AQP2 labeling to the plasma membrane compared to vehicle‐treated control tubules. However, there was no evidence of redistribution of AQP2 to the plasma membrane in response to roflumilast (*n* = 3).

### Neither roflumilast nor roflumilast *N*‐oxide increases osmotic water permeability in isolated perfused IMCD segments

**Figure 6:**
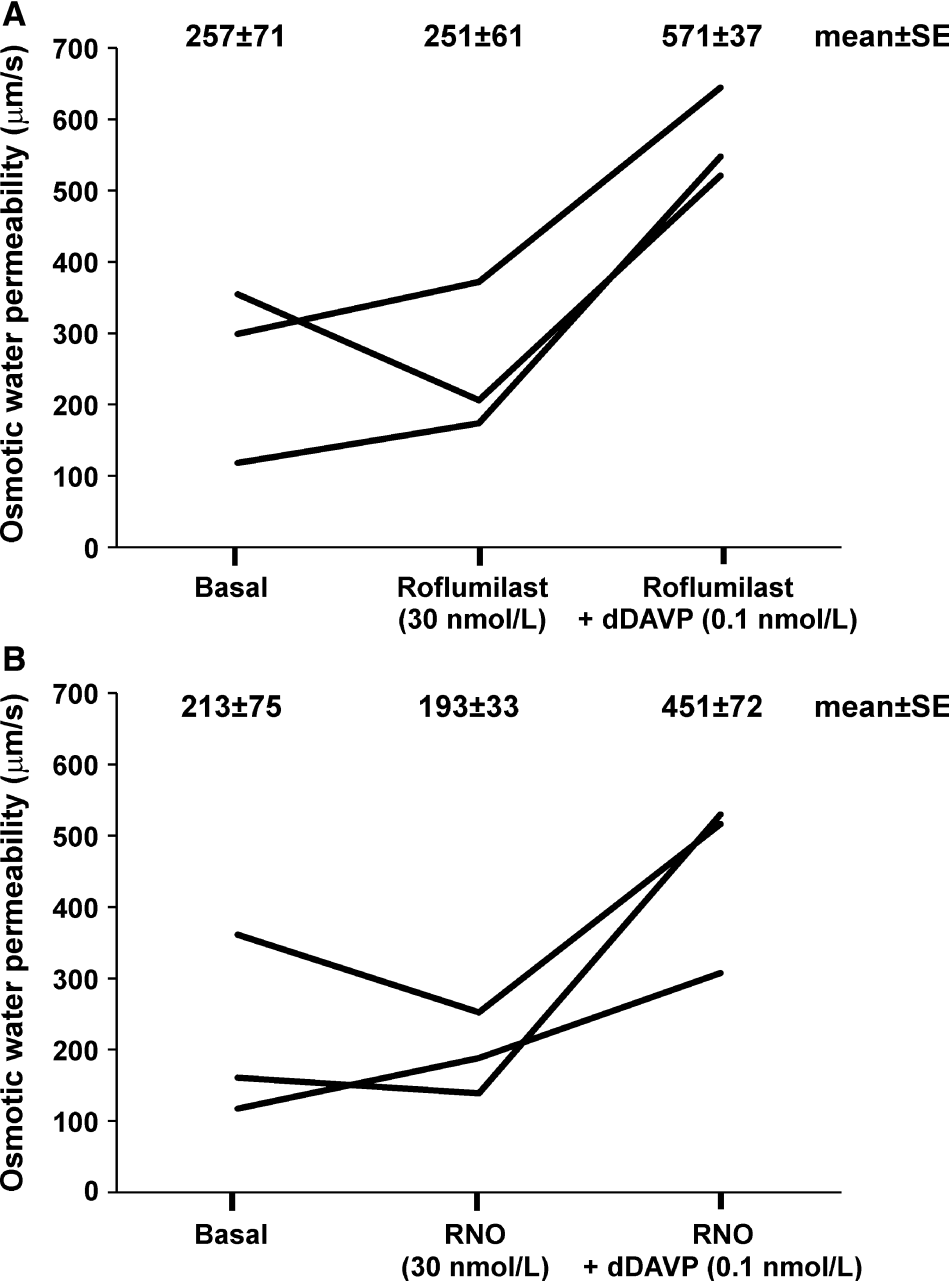
Osmotic water permeability (*P*_*f*_) measurements in microdissected IMCD segments. IMCD tubules were isolated and perfused for osmotic water permeability measurements in three 30‐min experimental periods: basal, roflumilast and roflumilast+dDAVP (upper panel) or basal, RNO and RNO+dDAVP (lower panel). Data from the same experimental period were pooled for statistical analysis. Water permeability measured at 30 nmol/L roflumilast or RNO alone was not statistically different from the basal condition, whereas in the presence of 0.1 nmol/L dDAVP, water permeability was significantly increased (*P* < 0.001, *n* = 6). IMCD, inner medullary collecting duct; RNO, roflumilast *N*‐oxide.

Despite the lack of effect of roflumilast on AQP2 phosphorylation or trafficking, it is conceivable that it could affect water permeability by channel gating or through effects on the basolateral water channels AQP3 or AQP4. Figure 6 shows results of osmotic water permeability measurements in isolated perfused IMCD segments with both roflumilast (upper) and RNO (lower). As can be visualized, neither agent increased osmotic water permeability. However, as expected, dDAVP increased water permeability demonstrating that the tubules are capable of responding.

## Discussion

This study tested the effect of roflumilast, a selective PDE4 inhibitor approved by FDA for the treatment of chronic obstructive lung disease, and its active metabolite RNO, on AQP2 regulation in the kidney IMCD. We can draw two main conclusions from this study: (1) although roflumilast alone has no effect on AQP2 phosphorylation, AQP2 trafficking, or water permeability, it enhanced the short‐term action of vasopressin to regulate AQP2 in the collecting duct; and (2) roflumilast is significantly more potent than IBMX, and therefore it might be a better choice than IBMX as a phosphodiesterase inhibitor for in vitro physiological studies in renal collecting duct cells. We consider these two conclusions in detail in the following.

### No effect of roflumilast alone

For roflumilast to be a good candidate for potential use in treating patients with X‐linked NDI, it should have demonstrable effects on AQP2 regulation in vitro. Results from this study provided no evidence that roflumilast alone can alter AQP2 phosphorylation, AQP2 localization, or water permeability in the collecting duct. Similar findings were reported by Stefan et al. (2007) in their studies on AQP2 regulation in primary cultures of renal IMCD cells using the selective PDE4 inhibitor rolipram, wherein they showed that rolipram alone does not alter the trafficking of AQP2 or increase osmotic water permeability. Likewise, Bichet et al. (1990) found no effect of rolipram when it was used in treating two male patients with congenital NDI. Taking our findings into consideration and the known toxicity of roflumilast in humans (Gross et al. 2010; Izquierdo and Aparicio 2010; Rabe 2010), we therefore concluded that roflumilast alone may not have a beneficial effect in X‐linked NDI. This conclusion does not exclude the possibility that roflumilast may be useful for patients with acquired NDI. It is also conceivable that roflumilast alone could have a regulatory effect on AQP2 gene expression over a longer time scale than tested in this study, as is the case with vasopressin (Terris et al. 1996). However, since both the short‐term trafficking effects of vasopressin and the long‐term effects on AQP2 protein abundance appear to be mediated by cAMP, it is unlikely that roflumilast would trigger an increase in AQP2 abundance with long term exposure.

Why did roflumilast fail to alter AQP2 phosphorylation or trafficking, when theoretically it should have increased cAMP signaling? One possibility is that the degradation of cAMP in the collecting duct is mediated by phosphodiesterases other than the four PDE4 proteins. Indeed, several other phosphodiesterases are expressed in collecting duct cells including *Pde1a*,* Pde1c, Pde3b*,* Pde5a, Pde6d*,* Pde7b*, and *Pde8a* (Lee et al. 2015; Uawithya et al. 2008). While the functionality of these phosphodiesterases varies from one isoform to another, their nucleotide substrate specificities often overlap. PDE1 is calcium/calmodulin‐activated with dual specificity for cAMP/cGMP substrates (Beavo 1995; Beavo et al. 1994; Dousa 1999; Kakiuchi et al. 1975; Klee and Vanaman 1982; Swinnen et al. 1989), PDE5 and PDE6 hydrolyze cGMP only (Beavo 1995; Beavo et al. 1994; Dousa 1999; Thompson 1991; Yanaka et al. 1998), PDE3 (Beavo 1995; Beavo et al. 1994; Degerman et al. 1997; Dousa 1999; Leroy et al. 1996; Maurice et al. 2014; Weishaar et al. 1985) is cGMP‐inhibited but hydrolyzes cAMP, while the isozymes PDE4 (Beavo 1995; Beavo et al. 1994; Conti et al. 2003; Davis 1984; Dousa 1999; Maurice et al. 2014; Schwabe et al. 1976; Swinnen et al. 1989; Wang et al. 1997), PDE7 (Beavo 1995; Bloom and Beavo 1996; Dousa 1999; Gardner et al. 2000; Michaeli et al. 1993), and PDE8 (Dousa 1999; Fisher et al. 1998; Hayashi et al. 1998; Soderling et al. 1998; Soderling and Beavo 2000) selectively hydrolyze cAMP. In addition, studies using isoform specific inhibitors have provided evidence for the expression of PDE1, PDE3, and PDE4 isoforms in renal collecting duct cells (Dousa 1999). Lee et al. has also provided further data on the phosphodiesterases expressed in the different segments of rat renal collecting duct through their deep sequencing studies (see Table 1). Therefore, it is plausible that the other PDE isoforms provide enough cAMP metabolizing activity to maintain low cAMP levels without PDE4 activity in the absence of a stimulus for cAMP production.

**Table 1:** Relative transcript abundance of PDE isoforms in rat kidney collecting duct

Gene	Description	Substrate	CCD	OMCD	IMCD
Pde1a	Calcium/	cAMP,	24.6	50.1	13.6
Pde1c	Calmodulin stimulated	cGMP	1.1	0	0
Pde3b	cGMP‐inhibited	cAMP	8.7	10.7	0
Pde4a	cAMP‐specific	cAMP	0	0	0.2
Pde4b			0.6	0	0
Pde4c			0	0	0.1
Pde5a	cGMP‐specific	cGMP	0.2	0	0.5
Pde6d	cGMP‐activated, cGMP‐specific	cGMP	32.9	0.2	62.4
Pde7b	cAMP‐specific	cAMP	9.9	0.4	0.4
Pde8a	cAMP‐specific	cAMP	14.4	15.9	3.3

PDE isoforms, description and substrate is adapted from Maurice et al. (2014) (Maurice). CCD, OMCD, and IMCD are collecting duct segments microdissected from the cortex, outer medulla and inner medulla of the kidney. Numbers are relative transcript abundances of PDE isoforms measured by a single tubule RNA‐seq technique (Lee et al. 2015) and expressed as the number of reads per million nucleotides in the whole genome.

This raises the question of a possible synergistic role between PDE4 and other cAMP‐selective metabolizing PDE isoforms. Interestingly, number of studies exploring the therapeutic applications of inactivating PDE4, as a means to alleviate different disease states, have revealed the potentiating effects of dual inhibition of PDE4 and one of the other cAMP‐hydrolyzing PDE isoforms, chiefly PDE3 and PDE8 (Banner and Press 2009; Giembycz and Newton 2011; Kraynik et al. 2013; Matera et al. 2014; Maurice et al. 2014; Mika et al. 2013; Palmer et al. 1998; Salari and Abdollahi 2012; Snyder et al. 2005; Zhao et al. 2008). One such study demonstrated that the simultaneous inhibition of PDE4 and PDE8 significantly increased steroidogenesis in Leydig cells (Demirbas et al. 2013; Golkowski et al. 2016; Shimizu‐Albergine et al. 2012). Inactivation of cAMP‐selective phosphodiesterase isoforms is not unique to this potential role. Indeed, several studies exploring the inhibition of cGMP‐specific PDEs using selective agents such as sildenafil and non‐cAMP/cGMP pathways have demonstrated cAMP‐independent ways of regulating AQP2 in renal collecting duct (Assadi and Sharbaf 2015; Bonfrate et al. 2015; Boone and Deen 2008; Boone et al. 2010; Bouley et al. 2000, 2005, 2008; Brown 2003; Cheung et al. 2016; Sanches and Volpini 2012). As such, this area of research holds potential promise for finding beneficial therapies for patients with X‐linked NDI and other types of diabetes insipidus.

Another possibility is that there was insufficient cAMP production in the absence of vasopressin to allow phosphodiesterase inhibition to cause a physiologically significant rise in cAMP to activate protein kinase A and other kinases necessary for AQP2 phosphorylation. Indeed, Stefan et al. (2007). had shown that inhibition of PDE4 alone with the selective PDE4 inhibitor rolipram did not produce measurable cAMP levels in whole cell assays of primary cultures of renal IMCD cells. They emphasize that whole cell assays do not necessarily assess cAMP changes in subcellular compartments. Theoretically, other methods of locally increasing the basal levels of cAMP could potentially enhance the effect of PDE4 inactivation on AQP2 regulation. We have previously demonstrated that another G protein‐coupled receptor, the prostaglandin E2 receptor EP4, was expressed at significant levels in mouse IMCD and EP4 agonist (ONO‐AE1‐329) was capable of increasing cAMP in mouse IMCD (Li et al. 2009). However, in this paper, ONO‐AE1‐329 produced no change in AQP2 phosphorylation, presumably because it does not affect cAMP in the subcellular compartment relevant to AQP2 phosphorylation which was not assessed in the previous paper (Li et al. 2009).

We also reasoned that the lack of responsiveness of rat IMCD to EP4 agonist (ONO‐AE1‐329) in the present study, in comparison with previous study (Li et al. 2009), could be related to species (previous in mouse; now in rat) or segment (previous Pf measurements in CCD, now in IMCD). Evidence supporting this speculation came from EP4 localization study in the rat kidney using RT‐PCR mRNA in which it was shown that EP4 is expressed very weakly in IMCD but is strongly expressed in the cortical collecting duct (Jensen et al. 2001). Gao et al. have also shown that EP4 protein abundance is very low in the mouse kidney medulla compared to other PGE2 receptor types, although its expression can be enhanced by water restriction to the animals (Gao et al. 2015). Additionally, Olesen et al. (2011) showed that another EP4 agonist (CAY10580) was capable of inducing AQP2 phosphorylation at pS256 in cortical tubule suspensions but it did not cause AQP2 targeting to the plasma membrane in native rat tissue. The absence of significant AQP2 phosphorylation changes in response to EP4 agonist (ONO‐AE1‐329) in the present study is thus consistent with these previous findings in rats. Investigations of the long‐term effect of EP4 agonist have, however, identified soluble (pro)renin receptor (sPRR), by interacting with frizzled‐8 (FZD8), as a downstream effector of EP4 or V2R‐induced AQP2 expression and antidiuretic response in primary cultures of rat IMCD (Lu et al. 2016; Wang et al. 2016). These findings open potential applications for the use of PDE4 inhibitor in combination with EP4 agonist in a long‐term response to enhance the water permeability response in the renal collecting duct.

### Roflumilast is significantly more potent than IBMX

Our dose–response studies showed that roflumilast at a range of concentrations from 3 to 3000 nmol/L enhances the effect of vasopressin to increase AQP2 phosphorylation at S264 and S269. Because S269 plays a critical role in AQP2 trafficking by inhibiting AQP2 endocytosis (Hoffert et al. 2008), this observation reinforces the conclusions of prior studies that PDE4 isoforms play an important physiological role in AQP2 regulation in the collecting duct (Coffey et al. 1991; Feraille et al. 2014; Sohara et al. 2006; Stefan et al. 2007; Wang et al. 2002). In contrast, IBMX only enhanced the vasopressin‐dependent AQP2 phosphorylation at 3000 nmol/L. Our results suggest that roflumilast is several orders of magnitude more potent than IBMX.

When compared to rolipram, roflumilast has a significantly higher affinity for PDE4, with an IC_50_ that is over a 1000‐fold less than rolipram (Maurice et al. 2014; Rabe 2011). And roflumilast's remarkable efficacy over rolipram has been highlighted by several studies on PDE4 inhibition (Dastidar et al. 2009; Davis et al. 2009; Ikari et al. 2013; Togo et al. 2009; Wollin et al. 2006; Wunder et al. 2013). More importantly, Thompson et al. (2002) have demonstrated through their studies that selective inhibition of PDE4 by high‐affinity agents are more effective in increasing cAMP levels than low‐affinity ones and nonselective inhibitors. Taking all these into account, we conclude that roflumilast may be a better choice than IBMX or rolipram for the conduct of physiological studies on AQP2 regulation, and could be useful in the treatment of other water transport disorders in the renal collecting duct.

## Conflict of Interest

No conflicts of interest, financial, or otherwise, are declared by the author(s).
